# Author Correction: Engineering monocyte/macrophage−specific glucocerebrosidase expression in human hematopoietic stem cells using genome editing

**DOI:** 10.1038/s41467-020-18044-0

**Published:** 2020-08-20

**Authors:** Samantha G. Scharenberg, Edina Poletto, Katherine L. Lucot, Pasqualina Colella, Adam Sheikali, Thomas J. Montine, Matthew H. Porteus, Natalia Gomez-Ospina

**Affiliations:** 1 Department of Pediatrics, Stanford University School of Medicine, Stanford, CA USA; 2 Gene Therapy Center, Hospital de Clinicas de Porto Alegre, Porto Alegre, Brazil; 3 Department of Pathology, Stanford University School of Medicine, Stanford, CA USA

**Keywords:** Genetic vectors, Targeted gene repair, CRISPR-Cas systems

Correction to: *Nature Communications* 10.1038/s41467-020-17148-x, published online 03 July 2020.

The original version of this Article contained errors in the abstract. In three instances within the abstract, the enzyme glucocerebrosidase was incorrectly referred to as its substrate glucocerebroside. The error has been corrected in the HTML and the PDF of the original version of the Article.

