# Supplementary material for: Engineering monocyte/macrophage−specific glucocerebrosidase expression in human hematopoietic stem cells using genome editing
Source: Nat Commun. 2020 Jul 3;11:3327. doi: 10.1038/s41467-020-17148-x (PMC7335164; doi:10.1038/s41467-020-17148-x)
Supplement: Supplementary file 1 — Supplementary Information [file 41467_2020_17148_MOESM1_ESM.pdf]

Supplementary Information:

Engineering monocyte/macrophage specific glucocerebrosidase expression in human hematopoietic stem cells using genome editing

Scharenberg et, al.

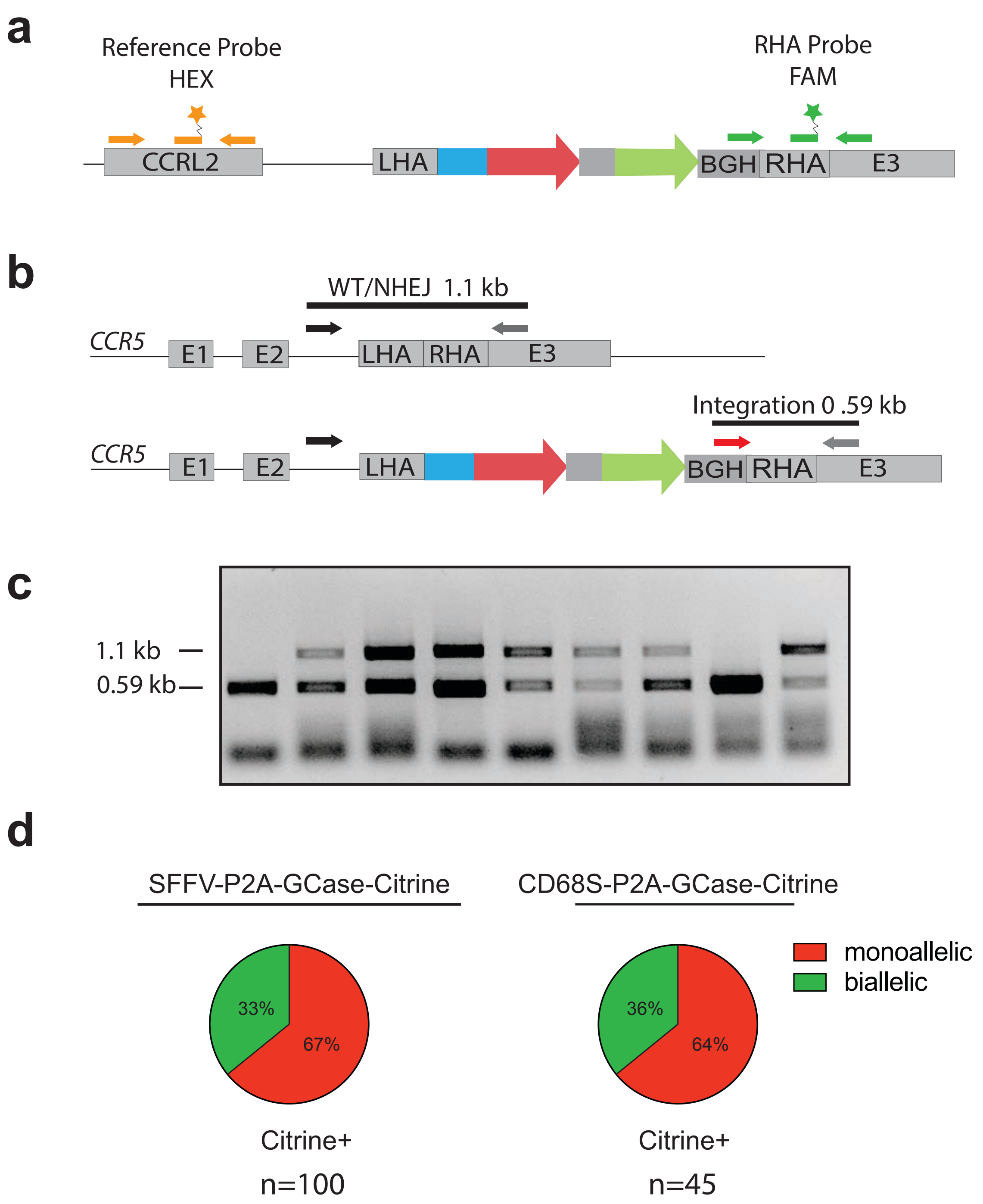

Supplementary Figure 1: Single cell-derived colony genotyping. a) Schematic of droplet-digital PCR assay to determine the percentage of targeted *CCR5* alleles in a given cell population. **b)** Schematic of three-primer PCR for single-cell genotyping. Wild-type or NHEJ-edited *CCR5* alleles yield a 1.1kb band and *CCR5* alleles with an integrated cassette (targeted) yield a 0.59 kb band **c)** Representative image of genotyping gel for 9 colonies depicting bands corresponding to wild-type/NHEJ and targeted *CCR5* alleles (n=100 colonies for the SFFV-GCase-P2A-Citrine and 45 colonies for the CD68S-GCase-P2A-Citrine vectors). **d)** Relative frequencies of mono- and bi-allelic editing of the SFFV-GCase-P2A-Citrine and CD68S-GCase-P2A-Citrine vectors. Source data are provided as a Source Data file.

a

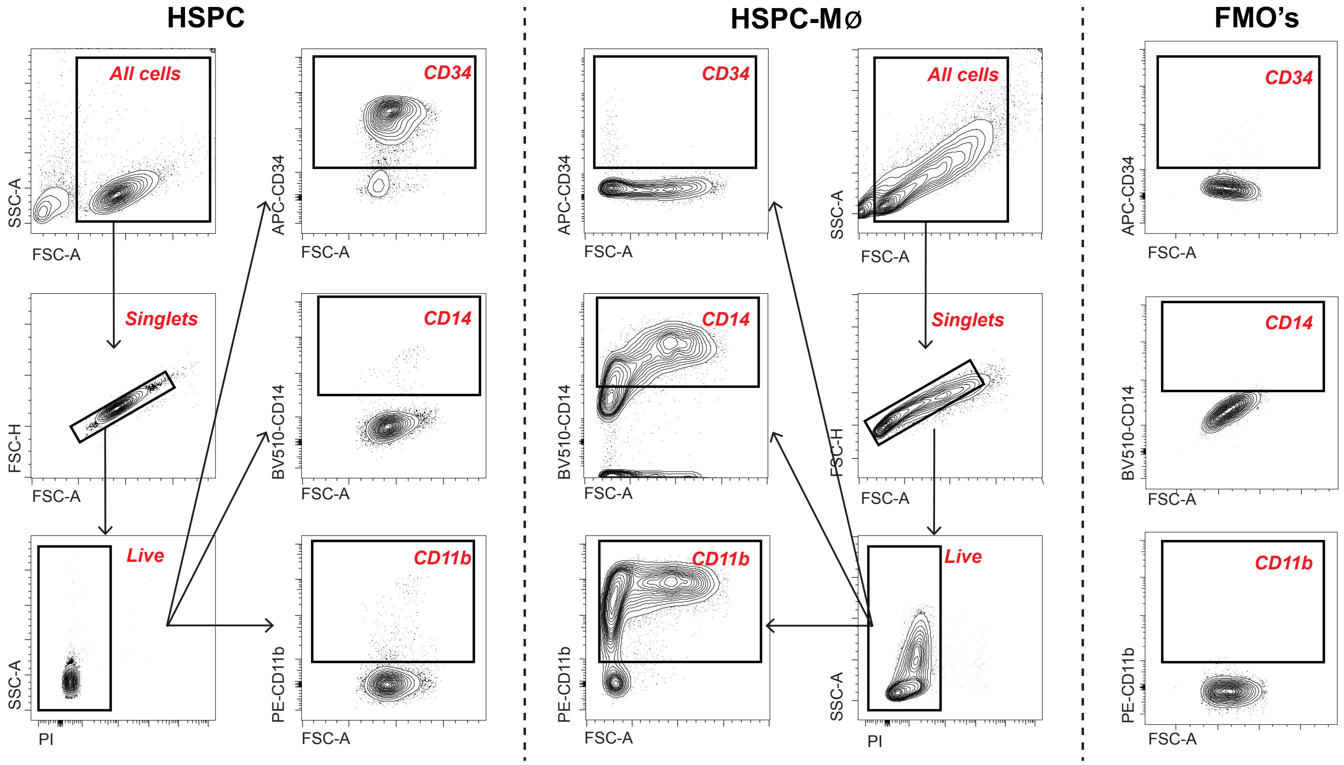

b

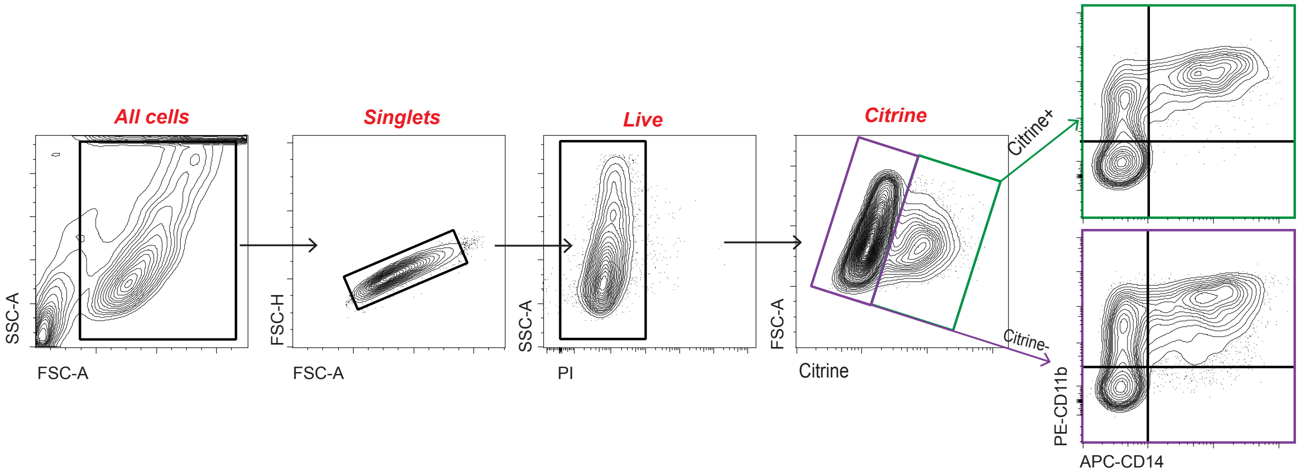

Supplementary Figure 2. Gating scheme. **a)** Flow cytometric quantification of human CD34+, CD14+, and CD11b+ cells in Mock-treated human HSPCs maintained in standard CD34+ cytokine media (HSPC) or media with M-CSF and GM-CSF to induce macrophage differentiation (HSPC-M∅). This strategy was used to quantify the data presented in Figure 2b. **b)** Gating scheme used to quantify flow cytometry data presented in 2d-e. FMO's are shown in the Figure 2e. Scatter axis differ between HSPC and HSPC-M∅ conditions as monocyte/ macrophage differentiation increases cell size and granularity.

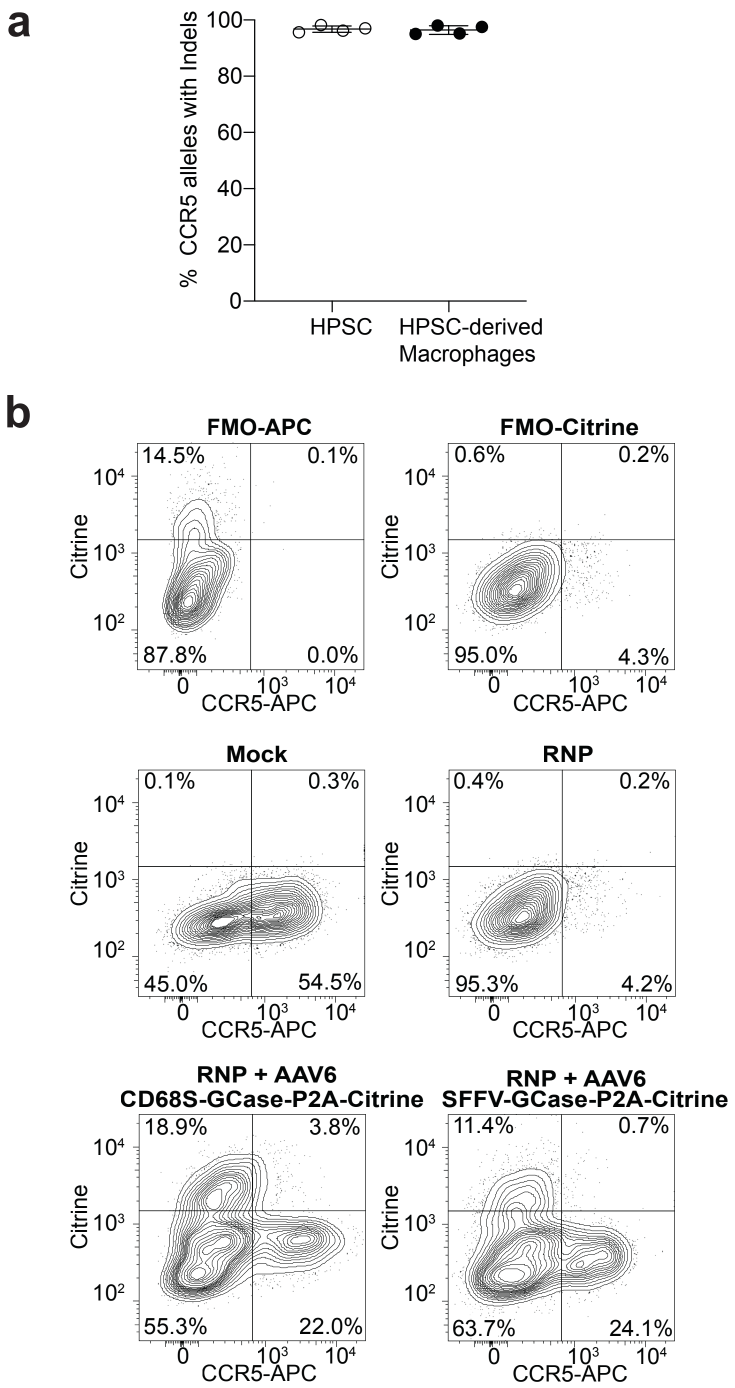

Supplementary Figure 3. CCR5 protein expression in targeted HSPCs. a) Indel frequency in CCR5 sgRNA + Cas9 (RNP) treated HSPCs cultured in either standard CD34+ cytokine media (open circles) or media with M-CSF and GM-CSF to induce macrophage differentiation (black circles) (n=4 independent human donor samples). **b)** Representative FACS plots showing Citrine and CCR5 (APC) expression in HSPC-derived macrophages. Top two panels: Fluorescence minus one (FMO) controls for Citrine and CCR5-APC. Middle two panels: CCR5 expression in Mock and RNP treated cells. Bottom two panels: Citrine vs. CCR5 expression in cells treated with RNP + SFFV-GCase-P2A-Citrine or CD68S-GCase-P2A-Citrine AAV's. Source data are provided as a Source Data file.

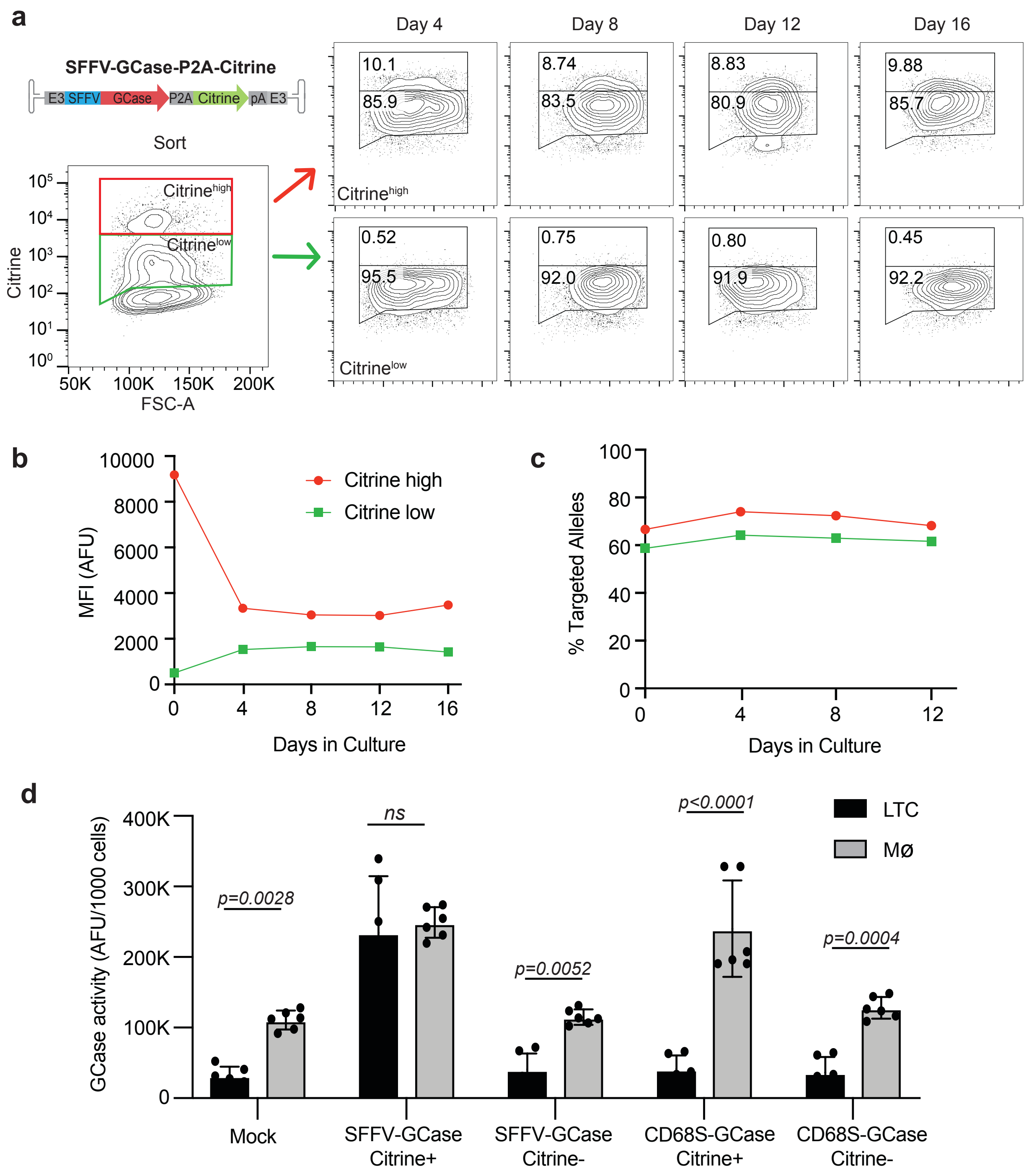

Supplementary Figure 4. Decline in Citrine expression in SFFV-GCase-P2A-Citrine+ HSPCs. **a)** Representative FACs plots showing Citrine expression over time in sorted populations of SFFV-GCase-P2A-Citrine-targeted HSPCs with high or low Citrine expression. **b)** Mean fluorescence intensity (MFI) for the same time points in sorted Citrine high and low cells. **c)** Percent of *CCR5* targeted alleles for the same time points in sorted Citrine high and low cells. **d)** GCase activity in HSPC and macrophage (Mø) cultures. Results are expressed as mean ± S.D. (n=6 biologically independent samples). All Comparisons were performed using two-way ANOVA followed by Bonferroni correction test for multiple comparisons. Source data are provided as a Source Data file.

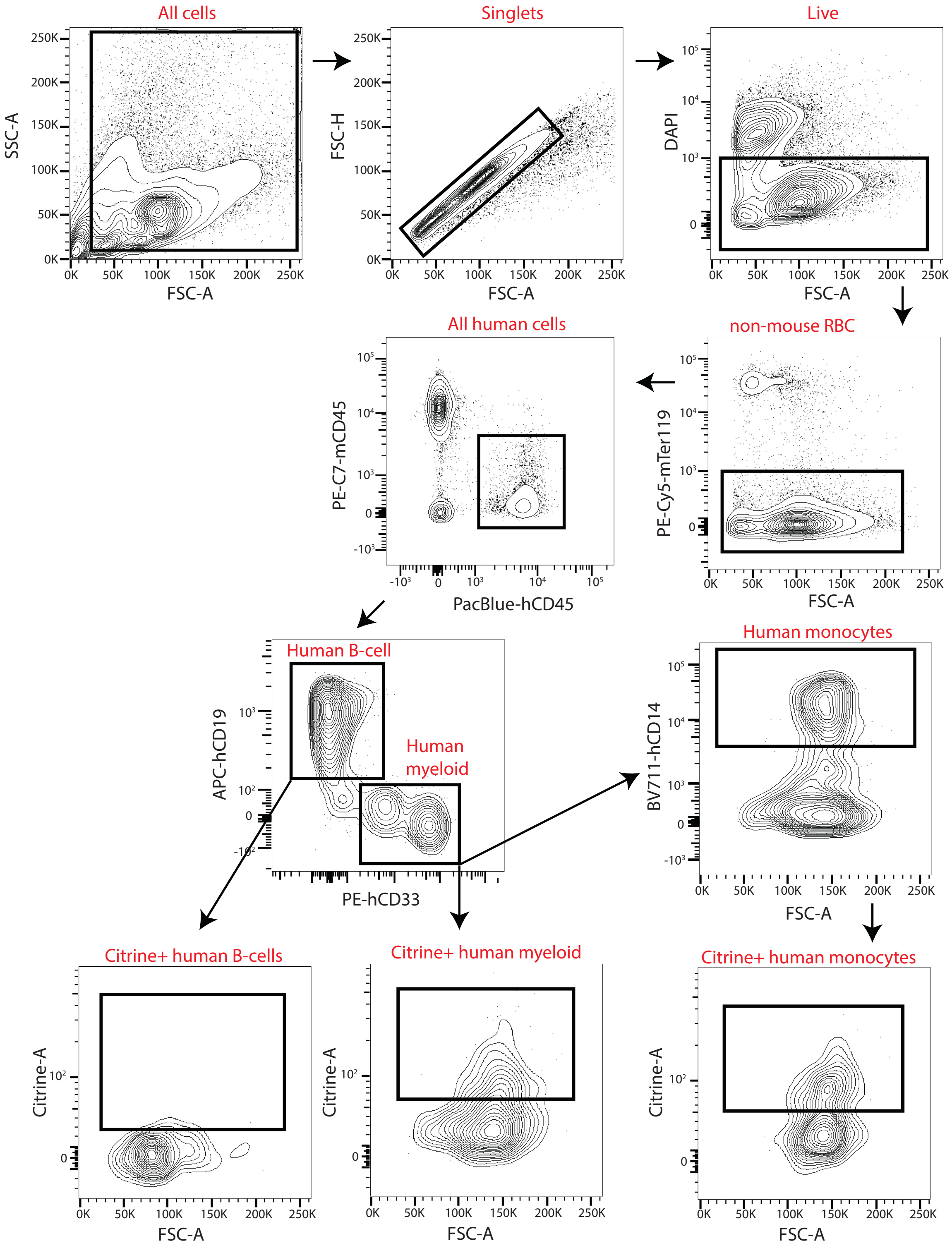

Supplementary Figure 5. Gating scheme for primary engraftment studies. Flow cytometric quantification of live, mouse RBCs, mouse CD45+, and human CD45+, CD19+ (B cells), CD33+ (myeloid cells), CD14+ (monocytes), and Citrine+ cells. Percent human engraftment was quantified as the percent hCD45+ cells over the combined mouse and human CD45+ population. This strategy was used to quantify the populations in Figure 4c, d, and f as well as 5a, c and d.

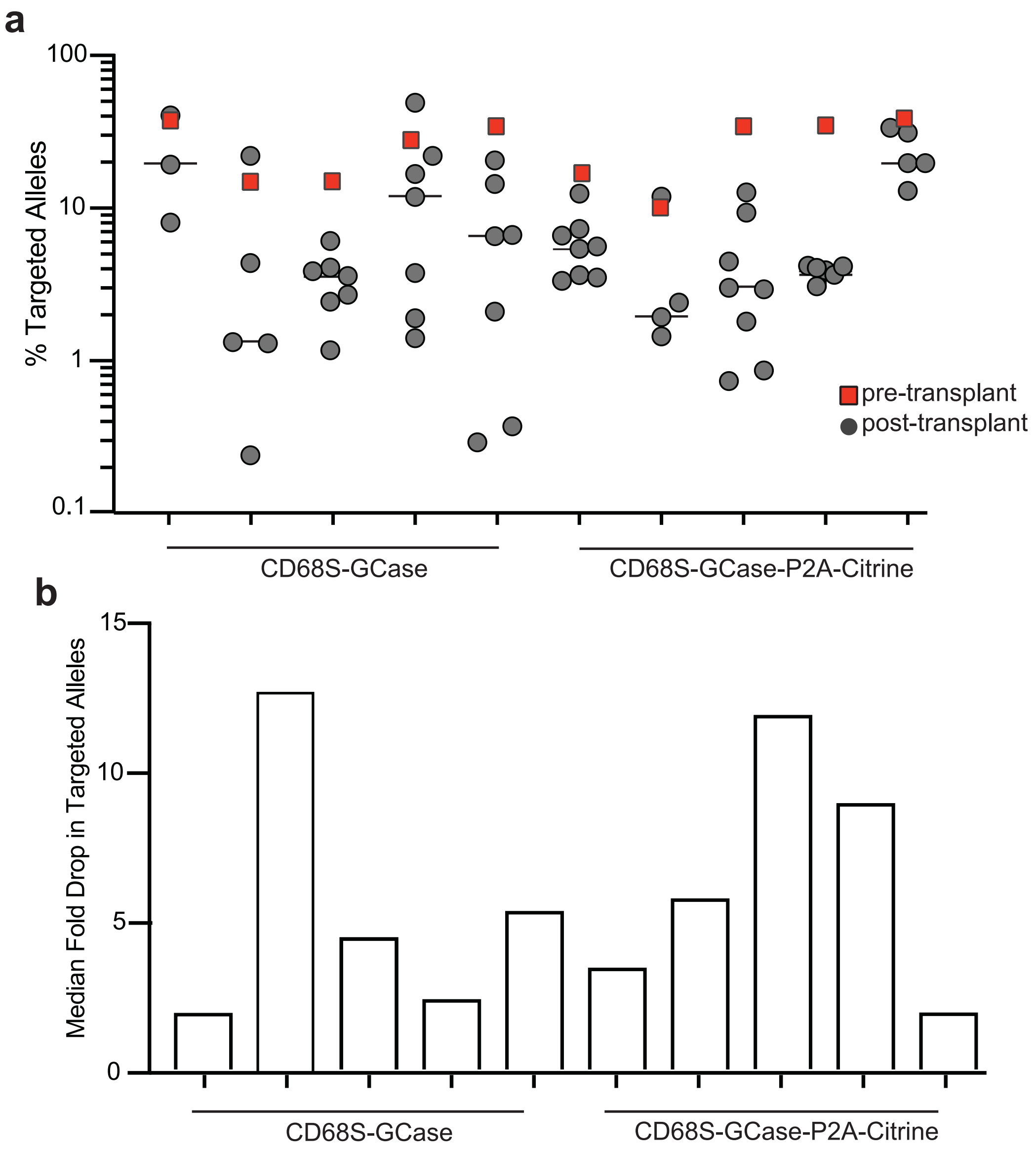

Supplementary Figure 6. Targeted CCR5 alleles before and 16-weeks post-transplantation. a) Targeted allele frequency from engrafted CD68S-GCase-targeted and CD68S-GCase-P2A-Citrine-targeted cells in 10 independent transplantation experiments (i.e., 10 different human donors). Red squares indicate modification allele fraction in HSPCs prior to transplantation, gray circles represent modification allele fraction in hCD45+ cells in primary engraftment mice in the bone marrow. Horizontal lines indicate median values. **b)** Median fold change in percent allelic modification with engraftment for each human cell donor. Source data are provided as a Source Data file.

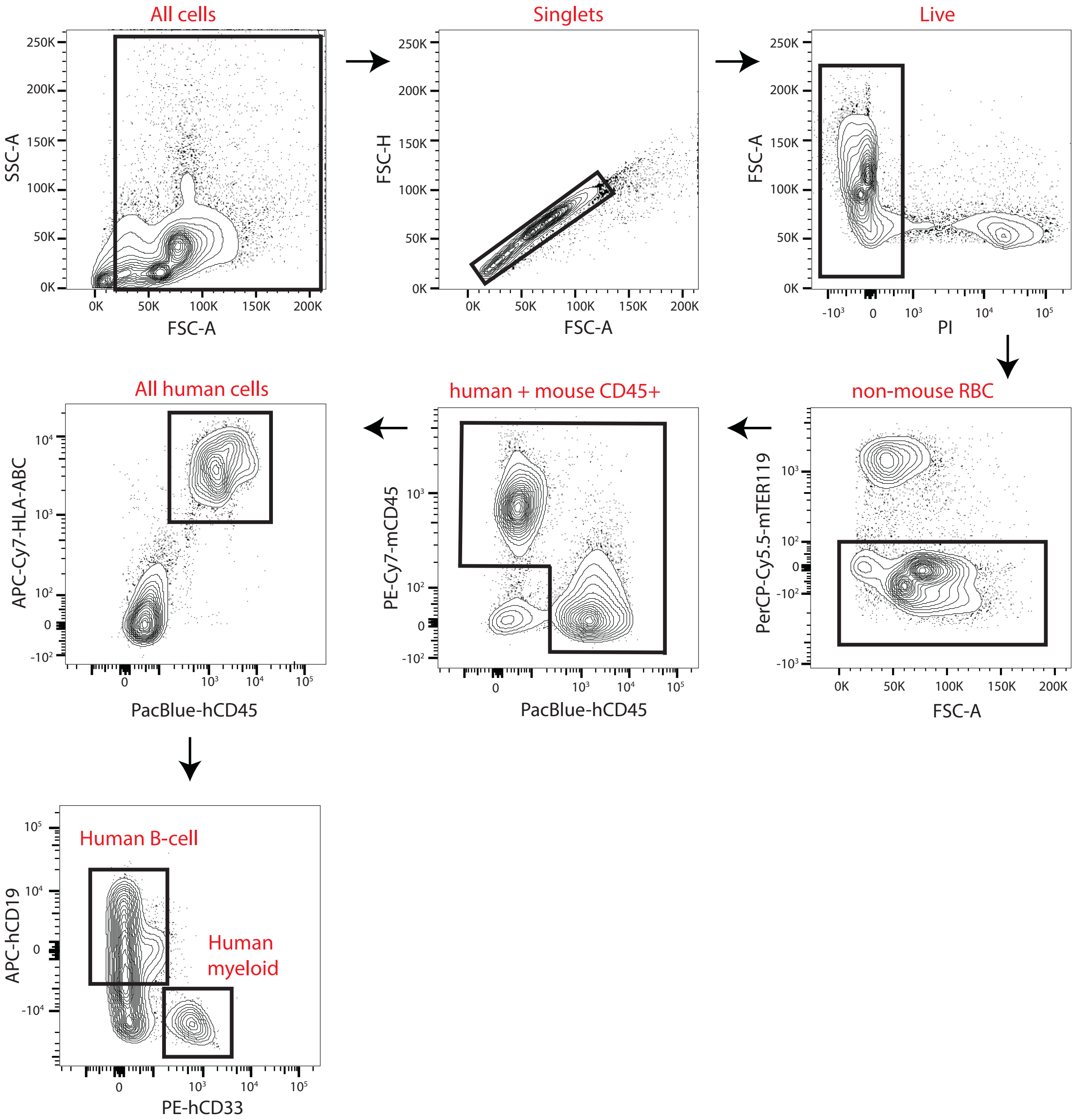

Supplementary Figure 7. Gating scheme for secondary engraftment studies. Flow cytometric quantification of live, mouse RBCs, mouse CD45+, and human CD45+, HLA-ABC+, CD19+ (B cells), and CD33+ (myeloid cells). Percent human engraftment was quantified as the percent hCD45+/HLA-ABC+ cells over the combined mouse and human CD45+ population. This strategy was used to quantify the populations in Figure 5b.

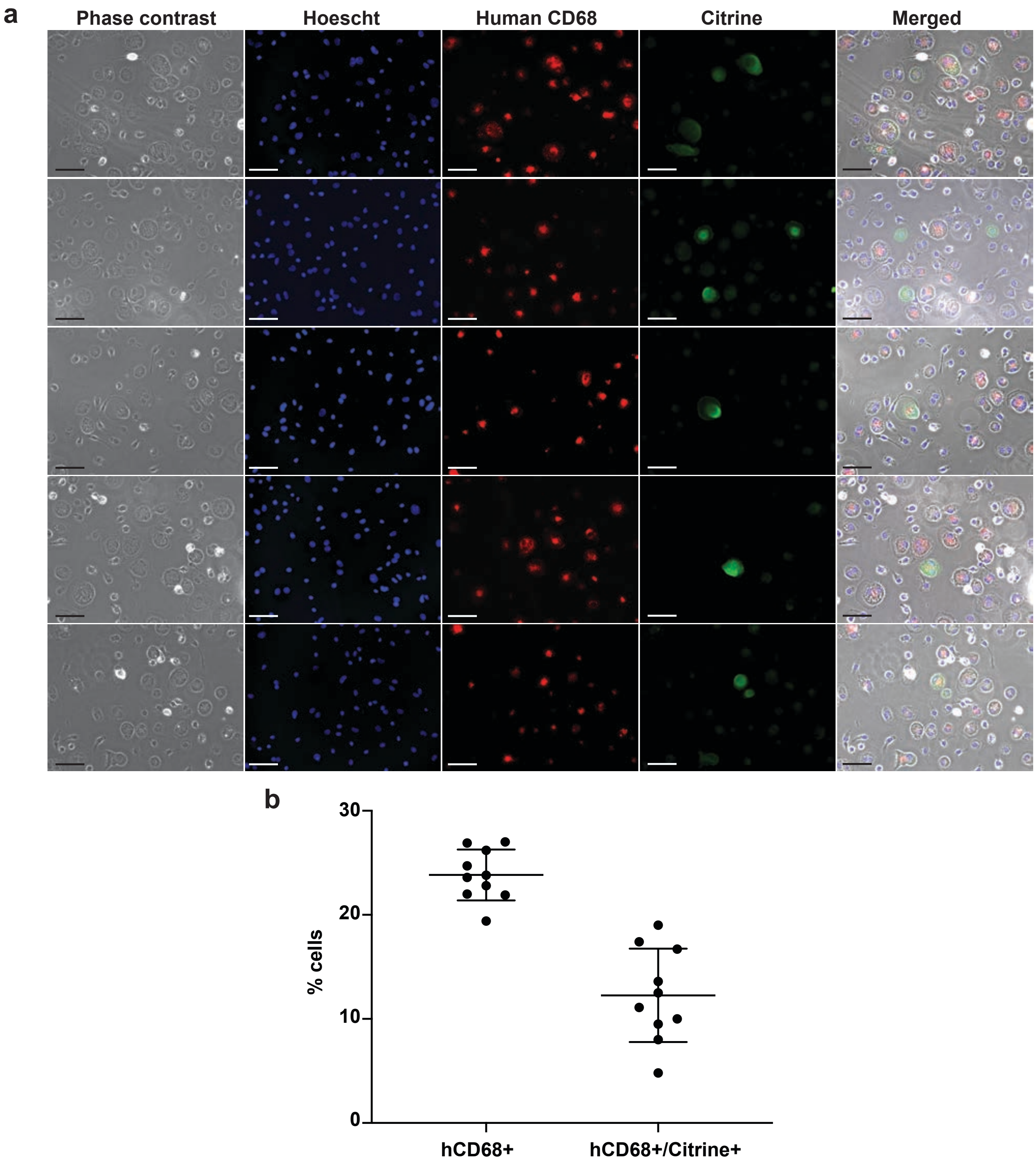

Supplementary Figure 8. Human GCase-targeted macrophages differentiated *in vivo*. a) Representative images showing phase contrast (gray), nuclei (Hoechst-blue), CD68 (red), Citrine (green) in CD68S-GCase-P2A-Citrine targeted macrophages. Most of the differentiation happened *in vivo* where targeted HSPCs engrafted and differentiated to human CD14+ promonocytes/monocytes. Cells were isolated from 10 different mice and cultured independently. Terminal differentiation was accomplished by addition of human M-CSF to isolated human CD14+ cells from mouse bone marrow. Each field corresponds to cells derived from a different mouse**.** Scale bar is 50µm. **b)** Percent CD68 and Citrine positive cells in these cultures (n=10 mice). Data shown as mean ± S.D. Source data are provided as a Source Data file.

Complete Primer list (All depicted as 5'->3')

**rAAV vector plasmid construction**	
CD68S-GCase-P2A-Citrine	
CCR5 5'HA_fwd	gtggccaactccatcactaggggttcctgcggccgctttcatgaattcccccaac
CCR5 5'HA_rev	tgggaacagtctagagaaggggacagtaagaag
CD68S_fwd	cttactgtccccttctctagactgttcccatagc
CD68S_rev	acttgaaaactccatggatccttcgaatcccctg
CCase cDNA_fwd	ggattcgaaggatccatggagttttcaagtccttccagag
CCase cDNA_rev	agtagctccgcttccctggcgacgccacaggta
P2A-Citrine-polyA_fwd	ctgtggcgtcgccagggaagcggagctactaac
P2A-Citrine-polyA_rev	agcatagtgagcccaactagtagcccaccgcatc
CCR5 3'HA_fwd	cggtgggctactagttgggctcactatgctgcc
CCR5 3'HA_rev	gtggccaactccatcactaggggttcctgcggccgcttgtagggagcccagaag
SFFV-GCase-P2A-Citrine	
CCR5 5'HA_fwd	gtggccaactccatcactaggggttcctgcggccgctttcatgaattcccccaac
CCR5 5'HA_rev	ttttatcggtctagagaaggggacagtaagaag
SFFV_fwd	cttactgtccccttctctagaccgataaaataaaagattttatttagtctcc
SFFV_rev	acttgaaaactccatggatccttcgaacccggg
CCase cDNA_fwd	gggttcgaaggatccatggagttttcaagtccttccagag
CCase cDNA_rev	agtagctccgcttccctggcgacgccacaggta
P2A-Citrine-polyA_fwd	ctgtggcgtcgccagggaagcggagctactaac
P2A-Citrine-polyA_rev	agcatagtgagcccaactagtagcccaccgcatc
CCR5 3'HA_fwd	cggtgggctactagttgggctcactatgctgcc
CCR5 3'HA_rev	gtggccaactccatcactaggggttcctgcggccgcttgtagggagcccagaag
CD68S-GCase	
CCR5 5'HA_fwd	gtggccaactccatcactaggggttcctgcggccgctttcatgaattcccccaac
CCR5 5'HA_rev	tgggaacagtctagagaaggggacagtaagaag
CD68S_fwd	cttactgtccccttctctagactgttcccatagc
CD68S_rev	acttgaaaactccatggatccttcgaatcccctg

CCase cDNA_fwd	ggattcgaaggatccatggagttttcaagtccttccagag
CCase cDNA_rev	cagcggctcgagctactggcgacgccacaggta
polyA_fwd	ctgtggcgtcgccagtagctcgagccgctgatc
polyA_rev	agcatagtgagcccaactagtagcccaccgcatc
CCR5 3'HA_fwd	cggtgggctactagttgggctcactatgctgcc
CCR5 3'HA_rev	gtggccaactccatcactaggggttcctgcggccgcttgtagggagcccagaag
**rAAV Titering by ddPCR**	
Target	AAV2 ITR
Fwd	GGAACCCCTAGTGATGGAGTT
Rev	CGGCCTCAGTGAGCG
Probe	/56FAM/CACTCCCTC/ZEN/TCTGCGCGCTCG/3IABkFQ/
**Measurement of cassette integration using ddPCR**	
Target	CCR5 3'HA
Fwd	GGGAGGATTGGGAAGACA
Rev	AGGTGTTCAGGAGAAGGACA
Probe	/56-FAM/AGCAGGCAT/ZEN/GCTGGGGATGCGGTGG/3IABkFQ/
Target	Reference CCRL2
fwd	GCTGTATGAATCCAGGTCC
rev	CCTCCTGGCTGAGAAAAAG
Probe	/5HEX/TGTTTCCTC/ZEN/CAGGATAAGGCAGCTGT/3IABkFQ/
**3-primer in-and-out PCR for colony genotyping**	
5'HA	CACCATGCTTGACCCAGTTT
polyA	CGCATTGTCTGAGTAGGTGT
3'HA	AGGTGTTCAGGAGAAGGACA
