# Supplementary material for: Engineering monocyte/macrophage−specific glucocerebrosidase expression in human hematopoietic stem cells using genome editing
Source: Nat Commun. 2020 Jul 3;11:3327. doi: 10.1038/s41467-020-17148-x (PMC7335164; doi:10.1038/s41467-020-17148-x)
Supplement: Supplementary file 3 — Reporting Summary [file 41467_2020_17148_MOESM3_ESM.pdf]

nature research

Corresponding author(s): Gomez-Ospina, Natalia

Last updated by author(s): 2020/05/24

Reporting Summary

Nature Research wishes to improve the reproducibility of the work that we publish. This form provides structure for consistency and transparency in reporting. For further information on Nature Research policies, see our Editorial Policies and the Editorial Policy Checklist.

Statistics

For all statistical analyses, confirm that the following items are present in the figure legend, table legend, main text, or Methods section.

n/a	Confirmed	
☐	☒	The exact sample size (n) for each experimental group/condition, given as a discrete number and unit of measurement
☐	☒	A statement on whether measurements were taken from distinct samples or whether the same sample was measured repeatedly
☐	☒	The statistical test(s) used AND whether they are one- or two-sided<br>*Only common tests should be described solely by name; describe more complex techniques in the Methods section.*
☒	☐	A description of all covariates tested
☐	☒	A description of any assumptions or corrections, such as tests of normality and adjustment for multiple comparisons
☐	☒	A full description of the statistical parameters including central tendency (e.g. means) or other basic estimates (e.g. regression coefficient) AND variation (e.g. standard deviation) or associated estimates of uncertainty (e.g. confidence intervals)
☐	☒	For null hypothesis testing, the test statistic (e.g. F, t, r) with confidence intervals, effect sizes, degrees of freedom and P value noted<br>*Give P values as exact values whenever suitable.*
☒	☐	For Bayesian analysis, information on the choice of priors and Markov chain Monte Carlo settings
☒	☐	For hierarchical and complex designs, identification of the appropriate level for tests and full reporting of outcomes
☒	☐	Estimates of effect sizes (e.g. Cohen's d, Pearson's r), indicating how they were calculated

Our web collection on statistics for biologists contains articles on many of the points above.

Software and code

Policy information about availability of computer code

Data collection	1. Snapgene 4.2 (GSL Biotech LLC) was used for planning, visualizing, and documenting cloning projects<br>2. QuantaSoft Analysis Pro v1.4 (Bio-Rad) was used to collect ddPCR data<br>3. BD FACSDIVA v8.0.1 (Becton Dickinson) software was included in the BD FACSaria II and was used to collect flow cytometry data<br>4. SoftMax Pro 7 for data Acquisition and analysis with the SpectraMax M3 plate reader in enzymatic studies.
Data analysis	1. FlowJo 10.6.2 (FlowJo, LLC) was used for analysis of flow cytometry data<br>2. QuantaSoft Analysis Pro v1.4 (Bio-Rad) was used to analyze ddPCR data<br>3. Images were quantified using imageJ 1.51 (open sourse)<br>4. Prism version 8.4 (GraphPad software) for graphing and statistical analysis<br>5. TIDE 2.0.1: Tracking Indels by Decomposition (TIDE) was used for quantifying INDEL rates (https://www.deskgen.com/landing/tide.html)

For manuscripts utilizing custom algorithms or software that are central to the research but not yet described in published literature, software must be made available to editors and reviewers. We strongly encourage code deposition in a community repository (e.g. GitHub). See the Nature Research guidelines for submitting code & software for further information.

Data

Policy information about availability of data

All manuscripts must include a data availability statement. This statement should provide the following information, where applicable:

- Accession codes, unique identifiers, or web links for publicly available datasets
- A list of figures that have associated raw data
- A description of any restrictions on data availability

The data that support the findings of this study are within the paper, its Supplementary Information files, or are available from the corresponding author N.G.-O.

upon request. All flow cytometry datasets in this study are available from Flowrepository, experiment number FR-FCM-Z2LQ. The source data underlying Figs. 1d-e, 2b and d, 3b-f, 4a-g, and 5a, b, and, as well as Supplementary Figs. 1d, 3a, 4b-d, 6a-b, and 8b are provided as a Source Data file

Field-specific reporting

Please select the one below that is the best fit for your research. If you are not sure, read the appropriate sections before making your selection.

- [x] Life sciences
- [ ] Behavioural & social sciences
- [ ] Ecological, evolutionary & environmental sciences

For a reference copy of the document with all sections, see nature.com/documents/nr-reporting-summary-flat.pdf

Life sciences study design

All studies must disclose on these points even when the disclosure is negative.

Sample size	No sample size calculations were performed. The number of animals used per transplant were determined by the number of cells successfully modified from a single human source divided by the intended dose. Sample size was increased by transplanting additional mice (different cell sources and different mouse litters). Final analysis is from a combination of these independent experiments.
Data exclusions	No datasets were excluded from analysis in this work.
Replication	The cells used in these experiments come from approximately 8 different human HSPC cell donors. Data is average of approximately 10 independent transplantation experiments in a total of 69 mice.<br>Characterization of the targeting efficiencies of the GCase cassettes was performed in 9 independent targeting experiments.<br>Generation of human GCase-macrophages from genome edited HSPCs and expression for the CD68 promoter was performed using 3 independent biological donor samples.<br>For the biochemical experiments of GCase activity in vitro 3 different human cell donors were analyzed in duplicate. For the biochemical experiments of GCase activity in vivo 3 different mice were analyzed in duplicate.<br>All attempts are replication were successful.
Randomization	Mice were randomly assigned to each experimental group and cage cohorts always consisted of mixed experimental conditions.
Blinding	Investigators were blinded to group allocation during data collection for all in vivo experiments. Mice were tagged with a code after transplantation. Data collection and analysis could not be done in a blinded fashion for many of the experiments using cells as the expression of the Citrine reporter would identify the constructs for FACS and imaging based assays.

Reporting for specific materials, systems and methods

We require information from authors about some types of materials, experimental systems and methods used in many studies. Here, indicate whether each material, system or method listed is relevant to your study. If you are not sure if a list item applies to your research, read the appropriate section before selecting a response.

Materials & experimental systems

n/a	Involved in the study	
☐	☒	Antibodies
☐	☒	Eukaryotic cell lines
☒	☐	Palaeontology and archaeology
☐	☒	Animals and other organisms
☐	☒	Human research participants
☒	☐	Clinical data
☒	☐	Dual use research of concern

Methods

n/a	Involved in the study	
☒	☐	ChIP-seq
☐	☒	Flow cytometry
☒	☐	MRI-based neuroimaging

Antibodies

Antibodies used	Flow cytometry<br>1. Anti-human CD34-APC (biolegend, #343509, clone 581, lot B260866)<br>2. Anti-human CD14-BV711 (BD Biosciences, #563373, clone MøP9, lot 8267739)<br>3. Anti-human CD14-BV510 (biolegend, #301842, clone M5E2, lot B237626)<br>4. Anti-Human CD14-APC (Invitrogen, # 17-0149-41, clone 61D3, lot 2149817)<br>5. Anti-human CD11b-PE (biolegend, #101208, clone M1/70, lot B279980)<br>6. Anti-human HLA-ABC APC-Cy7 (biolegend, #311426, clone W6/32, lot 267287)<br>7. Anti-human CD45-Pacific blue (biolegend, #368540, clone 2D1, lot B264394)<br>8. Anti-human CD19-APC (BD Biosciences, #555415, clone HIB19, lot 8296813 and 6312515)<br>9. Anti-human CD33-PE (BD Biosciences, #555450, clone WM53, lot 9010707)

10. Anti-mouse mter119 PE-Cy5 (Invitrogen, # 15-5921-83, clone ter-119, lot 2065639)
11. Anti-mouse CD45.1 PE-Cy7 (Invitrogen, # 25-0453-82, clone A20, lot 2055156)
12. Anti-human CCR5-APC (BD Biosciences, #556903, clone 2D7, lot 8332621)

Immunocytochemistry
1. Rabbit Anti-GFP (Abcam, ab290, polyclonal)
2. Mouse Anti-CD68 (Biolegend, #333801, clone Y1/82A, lot B265198)
3. Alexa Fluor® 488 Donkey anti-rabbit IgG (biolegend, #406416, Poly4064, lot B243796)
4. Goat anti-Mouse IgG (H+L) Cross-Adsorbed Secondary Antibody, Alexa Fluor 568 (Invitrogen/ThermoFisher A-11004, Lot 2090670)

Validation

All antibodies used herein were validated using several types of positive and negative controls. Positive controls included human peripheral blood mononuclear cells (PBMC's), mouse PBMC's, purified human T cells, and purified monocytes. Negative controls included fluorescence minus one controls, unstained samples, or cells from a species for the which the antibody is not supposed to show cross-reactivity. Species-specificity was verified in our single and mixed human-mouse samples.

Furthermore, all antibodies used here for flow cytometry have been previously reported and are routinely used. All vendors' (Biolegend, BD biosciences, eBiosciences/Invitrogen/ThermoFisher) report taking quality control measures to ensure that all antibodies sold are valid and reproducible. See https://www.biolegend.com/de-de/quality-control, and https://www.thermofisher.com/us/en/home/life-science/antibodies/invitrogen-antibody-validation.html for details on how each manufacturer validates their antibodies.

Eukaryotic cell lines

Policy information about cell lines

Cell line source(s)	293T cells from ATCC® CRL-3216
Authentication	Cells has typical epithelial morphology under the microscope. No other authentication procedures were performed.
Mycoplasma contamination	The cells were tested and were negative for Mycoplasma contamination.
Commonly misidentified lines (See ICLAC register)	None

Animals and other organisms

Policy information about studies involving animals; ARRIVE guidelines recommended for reporting animal research

Laboratory animals

Species: Mus musculus
Strains:
1) NSG: NOD.Cg-PrkdcscidIL2rgtmlWjl/Sz (NSG) mice were developed at The Jackson Laboratory.
2) NSG-SGM3: NOD.Cg-Prkdcscid Il2rgtm1Wjl Tg(CMV-IL3,CSF2,KITLG)1Eav/MloySzJ were described in Wunderlich et al, and Billerbeck et al45,46 and obtained from The Jackson Laboratory.
Age:
Mice were transplanted either in the neonatal period or at 6-8 weeks. Primary engraftment was measured 16 weeks post-transplantation, secondary transplants were analyzed after an additional 16 weeks (total 32 weeks).
Sex:
For engraftment studies, we transplanted 64 NSG and 5 NSG-SGM3 mice with roughly equal distribution of males and females.

Wild animals	This study did not involve wild animals.
Field-collected samples	This study did not involve samples collected from the field.
Ethics oversight	All experiments were performed in accordance with National Institutes of Health institutional guidelines and were approved by the University Administrative Panel on Laboratory Animal Care (IACUC 20565 and 33365).

Note that full information on the approval of the study protocol must also be provided in the manuscript.

Human research participants

Policy information about studies involving human research participants

Population characteristics	Human Cord blood was obtained through The Binns Program for Cord Blood Research Program and not by the investigators themselves. Eligible donors were expectant mothers scheduled to deliver at Lucile Packard Children's Hospital. Processed units were made available to Stanford researchers.

Recruitment	Expectant mothers provided informed consent before collection of the cord blood
Ethics oversight	The Program was approved by Stanford's IRB

Note that full information on the approval of the study protocol must also be provided in the manuscript.

Flow Cytometry

Plots

Confirm that:

- [x] The axis labels state the marker and fluorochrome used (e.g. CD4-FITC).
- [x] The axis scales are clearly visible. Include numbers along axes only for bottom left plot of group (a 'group' is an analysis of identical markers).
- [x] All plots are contour plots with outliers or pseudocolor plots.
- [x] A numerical value for number of cells or percentage (with statistics) is provided.

Methodology

Sample preparation	Cells were spun down, washed, and stained with propidium iodine or live/dead Blue Reactive Dye (ThermoFisher #L34961) for live/dead cell identification.
Instrument	Cells were analyzed by viability, Citrine and specific fluorophores using a BD FACSAria II flow cytometer (BD Biosciences).
Software	D FACSDIVA (Becton Dickinson) software was included in the BD FACSaria II and was used to collect flow cytometry data and for compensation. FlowJo 10.6 (FlowJo, LLC) was used for analysis.
Cell population abundance	For ddPCR 1,000-5,000 cells were sorted. For enzyme analysis of engrafted human cells 150,000 cells were sorted
Gating strategy	Gating strategies are shown in several supplementary files. Fluorescence minus one controls were used for gating and are included in most figures.<br>For Citrrine expression cells were gated based on FSC/SSC, doublet discrimination, live/dead, and Citrine.<br>For engraftment studies cells were gated based on FSC/SSC, doublet discrimination, live/dead, human and mouse CD45, human CD45 (and HLA-ABC positive in secondary transplants). Human B, and myeloid cells were identified using the human CD19, and CD33 antibodies. Monocytes were identified with a CD14 antibody.

- [x] Tick this box to confirm that a figure exemplifying the gating strategy is provided in the Supplementary Information.
